# Differential Effects of Vitamin C from Fruit and Vegetables Versus Supplements on the Risk of Frailty

**DOI:** 10.3390/nu17243876

**Published:** 2025-12-11

**Authors:** Seulgi Lee, Kirang Kim

**Affiliations:** Department of Food Science and Nutrition, Dankook University, Cheonan 31116, Republic of Korea; seulgilee@dankook.ac.kr

**Keywords:** frailty, fruit, vegetable, vitamin C, supplement

## Abstract

Background/Objectives: Frailty represents a critical aging-related condition, but evidence on how different sources of vitamin C relate to frailty risk remains limited. Thus, this study aimed to examine the relationship between frailty risk and sources of vitamin C intake (dietary, including fruit and vegetable (FV) vs. supplemental) among Korean adults. Method: We analyzed data from 9478 adults in the Korea National Health and Nutrition Examination Survey (KNHANES 2018–2019). Frailty was assessed using a modified Fried phenotype. A multivariable logistic regression model was used to calculate the odds ratios (ORs) and 95% confidence intervals (CIs) for frailty according to vitamin C intake source. Results: More than 60% of participants had inadequate FV intake. Significant associations were observed primarily in women. Increased FV intake (OR = 0.44, 95% CI = 0.264–0.731, comparing the highest intake group (fourth quartile, Q4) vs. the lowest intake group (first quartile, Q1)) for dietary vitamin C intake (OR = 0.60, 95% CI = 0.393–0.914, Q4 vs. Q1) and vitamin C intake from FV (OR = 0.54, 95% CI = 0.348–0.851, Q4 vs. Q1), was significantly associated with a lower risk of frailty. Women with inadequate FV intake had a higher risk of frailty (OR = 2.06, 95% CI = 1.34–3.16) compared to those with adequate intake, regardless of vitamin C supplement use. In contrast, vitamin C supplementation was not significantly associated with frailty risk in either men or women. Conclusion: A higher intake of FV and dietary vitamin C, but not supplemental vitamin C, was associated with a lower risk of frailty, particularly among women. These findings suggest that improving overall diet quality through increased FV consumption may be more effective for frailty prevention than relying on single-nutrient supplementation.

## 1. Introduction

As the global population ages, healthy aging, which encompasses a health span that extends physical and mental functions beyond life expectancy alone, is becoming a key goal of public health [1,2]. However, the most representative threat that impedes healthy aging is frailty [3,4]. Frailty is not a normal aspect of the aging process associated with increasing age, but rather a clinical syndrome characterized by an abnormally increased physical vulnerability to various stressors and a significant reduction in physiological reserve, making it a major factor that substantially elevates the risk of premature death [3,5].

Nutritional intervention is a key modifiable factor in the prevention and management of frailty in older adults [6,7], and there is evidence that frailty-related conditions, once developed, may still be reversible through nutritional interventions prior to the onset of frailty, and thus, nutritional intervention plays a critical role in aging-related conditions [8]. In particular, the role of antioxidant nutrients in alleviating oxidative stress and chronic inflammation, which are major pathophysiological mechanisms of senility, is being treated as important [9,10].

Among antioxidant nutrients, vitamin C is particularly relevant for frailty prevention due to its specific biological mechanisms. Vitamin C serves as an essential cofactor for collagen synthesis, which is critical for maintaining musculoskeletal integrity [11], and supports mitochondrial function for efficient energy metabolism [12]. Furthermore, its antioxidant properties may protect against sarcopenia by mitigating oxidative stress-induced muscle atrophy [11]. Given these roles, ensuring sufficient vitamin C intake is vital for aging populations. However, the intake of fruit and vegetables (FVs), the main source of vitamin C, currently falls short of recommended levels worldwide, including in Korea [13,14]. In contrast, the use of vitamin C supplements has increased markedly as a means to compensate for these nutritional inadequacies [14,15,16].

Previous studies have reported that the association between vitamin C and disease risk varies depending on its source. Specifically, dietary vitamin C intake has been linked to a reduced risk of cardiovascular disease and lung cancer, whereas vitamin C supplementation has not shown consistent evidence of conferring similar preventive effects [17,18,19]. This discrepancy may be attributed to the “food matrix” effect, where vitamin C in whole foods interacts synergistically with other bioactive compounds, such as phytochemicals and dietary fiber, potentially offering distinct health benefits compared to isolated nutrients found in supplements [20,21,22]. However, despite evidence that the health effects of vitamin C differ by source, there is limited research examining how such source-dependent differences are related to the risk of frailty. Based on these considerations, we hypothesized that the association between vitamin C intake and frailty risk would be source-dependent, potentially exhibiting distinct patterns for dietary versus supplemental intake. Therefore, this study aimed to examine the relationship between risk of frailty and different sources of vitamin C intake (total dietary vitamin C, vitamin C from FV, vitamin C from supplements and total vitamin C), and to evaluate whether these relationships differ by vitamin C source in Korean adults.

## 2. Subjects and Methods

### 2.1. Data and Participants

This study analyzed raw data from the Korea National Health and Nutrition Examination Survey (KNHANES) 2018–2019. The KNHANES is an annual, nationwide survey designed to assess the health and nutritional status of the Korean population using a random sampling methodology. Of the 16,102 total participants, 13,095 were adults aged 19 years or older. We applied the following exclusion criteria: (1) non-participation in the nutrition survey (*n* = 1620); (2) extreme energy intake (*n* = 205); (3) diagnosis of serious diseases (*n* = 1150); (4) pregnant or lactating women (*n* = 99); and (5) missing survey weight data (*n* = 543). Serious underlying diseases, defined as those diagnosed by a physician as reported in the health interview survey, included major cardiovascular diseases (stroke, myocardial infarction, and angina pectoris), liver diseases (liver cirrhosis), and malignant cancers (stomach, liver, colorectal, breast, cervical, lung, thyroid, and other cancers). After these exclusions, a total of 9478 participants were included in the final analysis.

### 2.2. Assessment of Frailty

For frailty assessment, modified criteria adapted from the Fried frailty phenotype were used [5]. Due to data availability in KNHANES, we adopted an operational definition that has been widely used and cited in previous studies analyzing KNHANES data [23,24]. The criteria were defined as follows: (1) Unintentional weight loss: Defined as unintentionally losing ≥3 kg in the past year; (2) Weakness: Defined according to the Asian Working Group for Sarcopenia (AWGS) 2019 criteria (handgrip strength < 28 kg for men and <18 kg for women) [25]; (3) Slow Gait Speed: Defined as responding “some problems in walking” or “confined to bed” to the mobility item of the Euro Qol 5-Dimensions (EQ-5D) questionnaire. (4) Exhaustion: Defined based on responses of “more than half the days” or “nearly every day” to the item “Feeling tired or having little energy” from the Patient Health Questionnaire-9 (PHQ-9) for 2018 survey data and affirmative response to the item “Felt low in energy” from the Health-related Quality of Life Instrument with 8 items (HINT-8) for 2019 survey data. (5) Low physical activity: Physical activity levels were assessed using the Global Physical Activity Questionnaire (GPAQ). Low physical activity was defined as <150 min of moderate-intensity physical activity per week or <75 min of vigorous-intensity physical activity per week. Participants who met three or more of these five criteria were classified into the frail group, while those who met fewer than three criteria were classified into the non-frail group.

### 2.3. FV Intake

FV intake was assessed using a 24 h dietary recall method. An adequate total intake of FV was defined as a minimum of 500 g per day, as suggested in the Korean Health Plan 2030 guidelines [26]. Participants were categorized based on this cutoff: individuals consuming less than 500 g/day were defined as the FV Inadequate (FVINA) group, while those consuming 500 g/day or more were defined as the FV Adequate (FVA) group.

### 2.4. Dietary and Supplemental Vitamin C Intake

Dietary vitamin C intake was assessed using the 24 h recall method. Vitamin C intake from supplements, including that from multivitamins, was determined using the supplement data obtained through the supplemental survey in the Korea National Health and Nutrition Examination Survey (KNHANES). Vitamin C intake was classified into the following categories: (1) total dietary vitamin C; (2) vitamin C from FV; (3) vitamin C from supplements; and (4) total vitamin C (diet plus supplements). To calculate the proportion of the inadequate vitamin C group, we classified subjects whose intake was below the Estimated Average Requirement (EAR) criteria for vitamin C (75 mg) as having inadequate vitamin C, based on the Dietary Reference Intakes for Koreans [27].

### 2.5. Other Variables

Sociodemographic factors were collected, including age, household type, frequency of eating alone, household income, education level, alcohol consumption, and smoking status. Age was categorized into three groups: young adults (19–39 years), middle-aged adults (40–64 years), and older adults (≥65 years). Household type was classified as ‘single-person household’ or ‘multi-person household’, and household income was categorized into quintiles. Education level was classified into four groups: ‘elementary school graduate or less’, ‘middle school graduate’, ‘high school graduate’, and ‘college graduate or higher’. The frequency of eating alone was categorized as ‘always eat alone’, ‘sometimes eat alone’, or ‘always eat with others’. For behavioral factors, participants who reported ‘smoked in the past but do not currently smoke’ were classified as non-smokers. Similarly, participants who reported ‘did not drink alcohol at all in the past year’ were classified as non-drinkers.

Health status and comorbidities were also assessed. Participants were defined as having diabetes mellitus if they had a fasting blood glucose (FBG) level ≥ 126 mg/dL, a hemoglobin A1c (HbA1c) level ≥ 6.5%, were taking anti-diabetic medication or insulin, or had a physician diagnosis of diabetes. Hypertension was defined as a systolic blood pressure (SBP) ≥ 140 mmHg, a diastolic blood pressure (DBP) ≥ 90 mmHg, or current use of anti-hypertensive medication. Finally, obesity classification was based on Body Mass Index (BMI, kg/m^2^) as follows: Underweight (<18.5), Normal weight (≥18.5 to <23), Overweight (≥23 to <25), and Obese (≥25). Diet quality was assessed using the Korean Healthy Eating Index (K-HEI), developed by the Korea Disease Control and Prevention Agency (KDCA). The K-HEI consists of 14 components encompassing adequacy, moderation, and energy balance, with a total score ranging from 0 to 100. A higher score indicates better overall diet quality [28]. Macronutrient intake was presented as the percentages of energy from carbohydrates, fat, and protein, calculated based on each participant’s total energy intake.

### 2.6. Statistical Analysis Methods

The KNHANES data were based on a complex, multi-stage sampling design that considered stratification, clustering, and systematic sampling. Therefore, all statistical analyses were performed by applying sample weights, stratification variables (strata), and cluster variables. Categorical variables are presented as weighted percentages (%), while continuous variables are presented as means with standard errors (SE). SE was reported instead of standard deviation (SD) to estimate the precision of the population mean while accounting for the complex survey design. The chi-square test was conducted to examine sociodemographic factors and the prevalence of meeting adequacy for FV intake and vitamin C intake (from various sources), and supplement use, according to frailty status. The independent samples *t*-test for complex samples was used to compare the intake of nutrients (including vitamin C) and FV according to frailty status. Multivariable logistic regression analysis was used to evaluate the risk of frailty, calculating odds ratios (ORs) and 95% confidence intervals (CIs). The models were adjusted for sociodemographic factors, lifestyle habits, prevalence of chronic diseases, total energy and protein intake, and the healthy eating index score. For this analysis, dietary vitamin C intake was energy-adjusted using the residual model. All statistical analyses were conducted using the Complex Samples module of SPSS version 30.0 for Windows (IBM SPSS Inc., Armonk, NY, USA), which accounted for the complex survey design. All tests were two-sided, and statistical significance was set at *p* < 0.05.

## 3. Results

### 3.1. The Characteristics of Subjects

The characteristics of subjects are described in Table 1. Of the 9478 participants, the prevalence of frailty was 2.9% in men and 5.6% in women. When compared by age group, the highest prevalence of frailty was observed in older adults, with 14.1% for older men and 22.3% for older women. When comparing characteristics by frailty status, the frail group among women had a nearly three-fold higher proportion of single-person households (27.4% vs. 9.1%, *p* < 0.001) and eating alone (31.3% vs. 11.4%, *p* < 0.001) compared to the non-frail group. Income and education levels were significantly lower in the frail group. Among lifestyle behaviors, the proportion of current drinkers was 62.0% in the frail men group and 43.9% in the frail women group. Energy intake was significantly lower in the frail group for both men and women. Regarding the macronutrient energy ratio, the frail group consumed significantly higher carbohydrates and lower protein and fat in both sexes. The average healthy eating index score was higher in the frail group than in the non-frail group, with no difference in men and a significant difference in women.

### 3.2. The Distribution of FV and Vitamin C Intake According to Frailty Status

The distribution of FV and vitamin C intake according to frailty status and sex is presented in Table 2. More than 60% of all groups did not meet the recommended FV intake, with significantly higher unmet proportion observed in the frail group. Regarding dietary vitamin C intake, over 70% of subjects in both groups consumed less than the EAR, showing no difference in the proportion between the two groups among men but a significantly higher proportion in the frail group among women. Vitamin C intake from FVs, a major source of vitamin C, showed no difference between the two groups in men. But the intake levels were significantly lower in the frail women. Vitamin C derived from FV accounted for approximately 70% of total dietary vitamin C, and this contribution was significantly higher in the frail group for both men and women. Regarding vitamin C supplementation, the proportion of supplement users was higher in the non-frail group compared to the frail group, with a significant difference in only men. The mean vitamin C intake from supplements was significantly higher in the non-frail group only among women. Total vitamin C intake (from both diet and supplements) was significantly higher in the non-frail group in both men and women. Regardless of frailty status, however, the proportion of individuals whose vitamin C intake—from diet as well as supplements—remained below the EAR was still substantial.

### 3.3. The Risk of Frailty According to Sources of Vitamin C Intake

After adjusting for confounding factors, the risk of frailty according to sources of vitamin C intake is presented in Table 3. The results were significant only in women, except for vitamin C intake from supplements. The result showed that for a 10 g increase in FV intake, the risk of frailty was decreased by 0.99 (OR = 0.99, 95% CI: 0.983–0.994). The risk was significantly lower in the highest intake group (Q4) of FV (OR = 0.44, 95% CI: 0.264–0.731, Q4 vs. Q1) as compared with the lowest intake group (Q1) among women. Dietary vitamin C (OR = 0.96 per 10 mg increase, 95% CI: 0.934–0.994; OR = 0.60, 95% CI: 0.393–0.914, Q4 vs. Q1) and vitamin C from FV (OR = 0.94, 95% CI: 0.905–0.976; OR = 0.54, 95% CI: 0.348–0.851, Q4 vs. Q1) were associated with a lower risk of frailty among women. However, vitamin C from supplements was not associated with the risk of frailty in either men or women. The total vitamin C intake from both diet and supplements showed an inverse association only in women, with a lower risk in the highest intake group (Q4) (OR = 0.61, 95% CI: 0.399–0.941, Q4 vs. Q1) as compared with the lowest intake group (Q1).

### 3.4. The Risk of Frailty by FV Adequacy and Vitamin C Supplement Use

Table 4 shows the combined effect of FV adequacy and vitamin C supplement use on the risk of frailty. The lower risk of frailty was significantly associated with adequate FV intake in only women, showing that the FVINA group had 2.04 times higher odds of frailty (OR = 2.04, 95% CI: 1.41–2.97) compared to the FVA group. There was no statistically significant association between supplement use and frailty risk in either men or women. Regarding the combined effect of FV intake level and vitamin C supplement use on frailty risk, women in the FVINA without supplements had a higher risk of frailty (OR = 2.06, 95% CI: 1.34–3.16), compared with those in the FVA without supplements. Regardless of FV adequacy, the risk in the supplement group did not differ from that in the FVA without supplements in either men or women.

## 4. Discussion

Epidemiological studies have consistently demonstrated an inverse association between FV intake and the risk of numerous chronic diseases, in part, due to their high vitamin C content [29,30]. Vitamin C has been advocated for the prevention and potential treatment of various health conditions because of its antioxidant and immune function. Recent studies have shown that the effect of vitamin C sources on the risk of chronic diseases such as lung cancer and cardiovascular disease depends on whether they are obtained from dietary sources or supplements [18,19,31]. Therefore, this study aimed to examine vitamin C intake and its effect on frailty risk according to intake of FV, which is a main source of dietary vitamin C and vitamin C supplement use, using nationally representative population data. Our findings found that higher intakes of FV and dietary vitamin C were significantly associated with a reduced risk of frailty, particularly among women, whereas supplemental vitamin C did not show a significant protective effect.

In this study, the proportion of inadequate FV intake was very high at over 60% for both men and women, regardless of frailty status. Notably, the frail group had a higher proportion of inadequate intake than the non-frail group. This is consistent with a previous study [13] showing that FV intake globally was not meeting recommended guidelines. Inadequate FV intake would directly lead to insufficient intake of vitamin C. This study showed that dietary vitamin C intake was also very low in both groups, with over 70% of participants consuming below the EAR. Vitamin C from FV accounted for approximately two-thirds of total dietary vitamin C intake. According to previous studies, the food sources contributing to dietary vitamin C intake were diverse. In particular, among younger individuals, the contribution from fruit intake was low, while a notable proportion of vitamin C was obtained from beverages and processed meats [32]. In the United States and the United Kingdom, the main source of vitamin C intake was fruit drinks such as fruit juice and smoothies, which was consistent with the eating patterns observed among young Koreans [32,33,34,35]. These findings highlight the need for public health policies aimed at increasing FV intake in the daily diet, including comprehensive interventions that promote higher FV consumption to prevent chronic diseases such as frailty.

In terms of FV and vitamin C intake from different sources in relation to the risk of frailty, FV and vitamin C from dietary factors were significantly associated with a lower risk of frailty, whereas vitamin C intake from supplements was not. Several studies have shown that higher FV consumption was associated with a lower frailty risk [36,37,38]. Antioxidants in FV may reduce oxidative stress and inflammation, thereby slowing aging and the development of frailty [39,40]. Specifically, phytochemicals and antioxidant nutrients, including vitamin C, which are major components of FV, may enhance immune function in older adults and help preserve mobility and physical function [41]. Previous studies have also reported that dietary vitamin C, including that obtained from FV, had an inverse relationship with chronic diseases such as cardiovascular disease, diabetes, and lung cancer [18,19,42,43].

In contrast, the effect of supplemental vitamin C on the risk of chronic diseases remains controversial. Several meta-analysis studies reported that vitamin C supplementation did not provide protective effects against chronic disease [18,42], and partial benefits were observed only when taken in combination with L-arginine or other antioxidants such as vitamin E [44,45]. Furthermore, excessive intake of vitamin C through high-dose supplements may reduce its antioxidant efficacy or even exert pro-oxidant effects [46]. In the present study, some participants exceeded the Tolerable Upper Intake Level (UL) of 2000 mg, with maximum supplemental intakes reaching 6000 mg and 6200 mg in non-frail men and women, respectively, compared to 1130 mg and 2000 mg in their frail counterparts. A recent Korean study similarly reported that there is no significant association between vitamin C supplementation and frailty among older adults [47]. In this study, for combined effects of FV adequacy and supplement use on frailty risk, inadequate FV intake was significantly associated with an increased risk of frailty regardless of vitamin C supplement intake, suggesting that FV intake level played a more critical role in determining frailty risk than vitamin C supplementation. Therefore, whole-food intake, including FV, should be addressed in public health messaging, considering the importance of fresh FV consumption not only for vitamin C intake but also for obtaining other bioactive compounds such as dietary fiber and phytochemicals.

Interestingly, in the analysis of FV and vitamin C intake by frailty status, no differences were observed among men, whereas significant differences were found only among women. These findings were consistent with the associations observed for frailty risk, which were significant only among women, aligning with previous studies reporting a sex-specific association between diet and frailty. Shibasaki et al. (2019) and Xu et al. (2021) reported that the association between dietary intake, including FV and frailty, was stronger in women than in men [48,49]. Several biological factors may explain these sex differences. First, women have higher requirements for micronutrients essential for skeletal maintenance and muscle function and are at greater risk of osteoporosis and fractures, which are closely linked to frailty [50,51,52,53]. As a result, the impact of specific dietary factors may be more pronounced in women than in men. Second, hormonal changes and chronic inflammation may also contribute. Postmenopausal women are more likely to experience chronic low-grade inflammation characterized by elevated C-reactive protein (CRP) and interleukin-6 (IL-6) due to decreased sex hormone levels [54]. This inflammation accelerates protein catabolism and sarcopenia, making inadequate intake of nutrients such as vitamin C potentially more impactful in women. These findings suggest that the development of tailored nutritional strategies, particularly for older women, may help inform public health recommendations aimed at preventing frailty.

This study has several limitations. First, the cross-sectional design prevents the establishment of causality. Specifically, there is a possibility of reverse causation, where frail individuals may consume fewer fresh foods (and thus less vitamin C) due to physical limitations or reduced access, rather than low vitamin C intake leading to frailty. Second, the dietary intake data were based on a single 24 h recall, which may not accurately reflect habitual or long-term intake. This inherent limitation can lead to random misclassification, which may attenuate or distort the observed associations between vitamin C intake and frailty. Third, the use of modified frailty proxies (PHQ-9, HINT-8 for exhaustion, AWGS thresholds for weakness, and EQ-5D mobility for slowness) may limit comparability with studies using direct assessments. We explicitly acknowledge that this is an operational definition adapted for the KNHANES dataset rather than a clinically validated diagnostic tool. However, similar operational definitions utilizing KNHANES variables have been implemented in previous research [23,24], supporting the feasibility of this approach. Finally, as this study was conducted exclusively on Korean adults, the findings may not be generalizable to other ethnic groups or populations with different dietary cultures and genetic backgrounds. Despite these limitations, this study has several strengths. It utilized data from the Korea National Health and Nutrition Examination Survey (KNHANES), which is a nationally representative dataset. Furthermore, the study is significant in that it specifically identified the effects of the different sources of vitamin C (diet vs. supplements) on the risk of frailty, rather than focusing solely on total vitamin C intake.

## 5. Conclusions

In conclusion, this study demonstrated that higher intake of FV and dietary vitamin C, but not supplemental vitamin C, was associated with a lower risk of frailty, particularly among women. In addition, women with inadequate FV intake had a higher risk of frailty compared to those with adequate intake, regardless of vitamin C supplement use, underscoring the critical role of whole-food sources rather than isolated nutrient supplementation. These findings highlight the importance of promoting sufficient consumption of FV as part of public health strategies to support healthy aging and prevent frailty. Future research is needed to explore the biological mechanisms underlying sex-specific associations and verify these relationships through longitudinal or interventional study designs.

## Figures and Tables

**Table 1:** General characteristics of subjects.

	Men		*p*	Women		*p*
	Non-Frail (*n* = 3565)	Frail (*n* = 163)		Non-Frail (*n* = 4738)	Frail (*n* = 382)	
**Total ^(1)^**	97.1 (0.29)	2.9 (0.29)	<0.001	94.4 (0.37)	5.6 (0.37)	<0.001
**Age group ^(1)^**						
Young adults	99.3 (0.23)	0.7 (0.23)	<0.001	98.8 (0.33)	1.2 (0.33)	<0.001
Middle aged adults	98.2 (0.38)	1.8 (0.38)		97.2 (0.37)	2.8 (0.37)	
Old adults	85.9 (1.44)	14.1 (1.44)		77.7 (1.49)	22.3 (1.49)	
**Household type**						
Single	10.5 (0.87)	13.1 (2.84)	0.352	9.1 (0.6)	27.4 (2.62)	<0.001
Living together	89.5 (0.87)	86.9 (2.84)		90.9 (0.6)	72.6 (2.62)	
**Meals with someone**						
Eating alone	10 (0.63)	26.1 (4.04)	<0.001	11.4 (0.54)	31.3 (2.92)	<0.001
Eating together sometimes	39.5 (1.06)	29.6 (4.34)		46.6 (0.9)	32.5 (3.2)	
Eating together always	50.5 (1.15)	44.2 (4.8)		42 (0.95)	36.2 (3.14)	
**Household income**						
Quintile 1 (lowest)	7.4 (0.56)	44.8 (4.12)	<0.001	9.6 (0.59)	43 (3.16)	<0.001
Quintile 2	14.8 (0.86)	28.6 (4.2)		17.3 (0.8)	25.1 (2.69)	
Quintile 3	21.5 (0.9)	5.8 (2.07)		21.4 (0.78)	11.6 (2.05)	
Quintile 4	27.4 (1)	9.8 (2.95)		25.1 (0.8)	11.6 (2.19)	
Quintile 5 (highest)	28.8 (1.25)	11 (3.49)		26.5 (1.1)	8.7 (1.74)	
**Education level**						
Elementary school graduate or less	6.2 (0.43)	35.4 (3.98)	<0.001	13.1 (0.66)	65.5 (3.04)	<0.001
Middle school graduate	6.3 (0.48)	19.4 (3.46)		7.4 (0.47)	10.5 (1.75)	
High school graduate	38.4 (1.02)	30 (4.47)		35.6 (0.9)	15 (2.27)	
College graduate or more	49.1 (1.23)	15.3 (3.95)		43.9 (1.12)	9 (1.98)	
**Alcohol consumption**						
None	12.6 (0.71)	38 (4.57)	<0.001	27.5 (0.75)	56.1 (2.95)	<0.001
Yes	87.4 (0.71)	62 (4.57)		72.5 (0.75)	43.9 (2.95)	
**Smoking status**						
None	65.8 (0.93)	61.1 (4.84)	0.336	93.5 (0.51)	95.3 (1.46)	0.3057
Yes	34.2 (0.93)	38.9 (4.84)		6.5 (0.51)	4.7 (1.46)	
**Diabetes**						
No	88.6 (0.62)	70.7 (4.76)	<0.001	92 (0.46)	70.3 (2.67)	<0.001
Yes	11.4 (0.62)	29.3 (4.76)		8 (0.46)	29.7 (2.67)	
**Hypertension**						
No	72.9 (0.89)	54.5 (4.42)	<0.001	78.3 (0.75)	37.6 (2.78)	<0.001
Yes	27.1 (0.89)	45.5 (4.42)		21.7 (0.75)	62.4 (2.78)	
**Obesity**						
Underweight	2.2 (0.29)	9.8 (2.96)	<0.001	6.1 (0.47)	7.2 (1.57)	<0.001
Normal	29.9 (0.89)	34.7 (4.44)		49.8 (0.88)	31.6 (2.82)	
Overweight	25.8 (0.9)	25.5 (4.5)		18.2 (0.61)	23.4 (2.62)	
Obesity	42.1 (0.92)	29.9 (4.82)		25.9 (0.78)	37.8 (2.89)	
**Nutrient intake**						
Total energy (kcal)	2132.7 ± 16.07	1721.1 ± 69.98	<0.001	1624.7 ± 11.61	1413.7 ± 34.55	<0.001
Total carbohydrate (%E) ^(2)^	61.1 ± 0.23	68.6 ± 1.41	<0.001	62.5 ± 0.22	70.5 ± 0.71	<0.001
Total protein (%E) ^(2)^	16.1 ± 0.11	14 ± 0.35	<0.001	15.2 ± 0.09	13.5 ± 0.22	<0.001
Total fat (%E) ^(2)^	22.8 ± 0.19	17.4 ± 1.25	<0.001	22.3 ± 0.17	16 ± 0.57	<0.001
**Health eating index**	59.4 ± 0.27	59.5 ± 1.16	0.943	61.9 ± 0.25	63.9 ± 0.62	0.003

All analyses were conducted using sampling weights to reflect the complex sample design; data are presented in tables as weighted % (SE). All tables present data as weighted mean ± SE for continuous variables. *p*-values were obtained from the Chi-square test for categorical variables and the independent *t*-test for complex samples for continuous variables. ^(1)^ This variable indicates the distribution between non-frail and frail groups. ^(2)^ %E indicates the percentage of nutrient intake relative to individual total energy intake.

**Table 2:** The distribution of FV and vitamin C intake according to frailty status.

	Men			Women		
	Non-Frail	Frail	*p*	Non-Frail	Frail	*p*
FV intake level ^(1)^						
g (mean ± SE)	452.1 ± 6.25	395.2 ± 24.41	0.028	413.3 ± 5.34	350.1 ± 12.34	<0.001
Inadequacy	65.7 (0.87)	73.1 (4.19)	0.113	70.8 (0.79)	80.0 (2.38)	0.001
Dietary vitamin C intake ^(2)^						
mg (mean ± SE)	69.3 ± 2.5	53.7 ± 9.1	0.098	61.4 ± 1.26	44.2 ± 2.46	<0.001
<EAR %(SE)	73.9 (0.95)	81.4 (4.13)	0.114	74 (0.84)	82.2 (2.23)	0.001
Vitamin C intake derived from FV ^(3)^						
mg (mean ± SE)	40.8 ± 0.99	38.6 ± 6.86	0.745	42.7 ± 1.04	31.2 ± 1.88	<0.001
FV contribution (%) to dietary vitamin C intake	67.9 ± 0.58	74.4 ± 2.18	0.004	69.1 ± 0.48	72.8 ± 1.59	0.026
<EAR %(SE)	87.3 (0.67)	90.7 (2.97)	0.320	84.2 (0.74)	90.2 (1.66)	0.003
Supplemental vitamin C intake in users ^(4)^						
supplement users %	24.4 (0.8)	13.2 (3.2)	0.006	28.9 (0.8)	25.7 (2.6)	0.251
mg (mean ± SE)	398.9 ± 26.51	456.8 ± 117.27	0.632	448.5 ± 20.24	317.5 ± 55.28	0.026
mg (median (min-max))	140 (0.01–6000)	140 (0.19–1130)		140 (0.01–6200)	100 (0.03–2000)	
Dietary and supplemental vitamin C intake ^(5)^						
mg (mean ± SE)	166.7 ± 7.97	113.9 ± 25.01	0.044	190.8 ± 7.17	125.7 ± 16.7	<0.001
<EAR %(SE)	60.6 (1.03)	73.4 (4.52)	0.012	57.7 (0.9)	67.5 (2.78)	0.001

All analyses were conducted using sampling weights to reflect the complex sample design. *p*-values were obtained from the Chi-square test for categorical variables and the independent *t*-test for complex samples for continuous variables. EAR, Estimated Average Requirement; FV, fruits and vegetables. The EAR for vitamin C is 75 mg according to the Dietary Reference Intakes for Koreans. ^(1)^ FV intake level was categorized as “inadequate” if the total intake of fruits and vegetables was <500 g. ^(2)^ It represents the weighted mean ± SE intake of vitamin C from diet only. ^(3)^ It represents the weighted mean ± SE intake of vitamin C from FV only. ^(4)^ It represents the weighted mean ± SE intake of vitamin C from supplements only. ^(5)^ It represents the weighted mean ± SE of the total intake of vitamin C from both diet and supplements.

**Table 3:** The risk of frailty according to sources of vitamin C intake.

	Men			Women		
	OR	95% CI		OR	95% CI	
FV intake						
g ^(2)^	1.00	0.988	1.005	0.99	0.983	0.994
Q1	Reference			Reference		
Q2	1.49	0.840	2.644	0.86	0.582	1.272
Q3	1.18	0.611	2.267	0.76	0.510	1.140
Q4	1.11	0.497	2.495	0.44	0.264	0.731
Dietary vitamin C intake ^(1)^						
mg ^(2)^	1.01	0.989	1.031	0.96	0.934	0.994
Q1	Reference			Reference		
Q2	1.16	0.665	2.008	0.72	0.455	1.125
Q3	1.22	0.710	2.112	0.78	0.518	1.158
Q4	1.08	0.542	2.139	0.60	0.393	0.914
Vitamin C intake derived from FV ^(1)^						
mg ^(2)^	1.04	0.975	1.109	0.94	0.905	0.976
Q1	Reference			Reference		
Q2	1.03	0.578	1.838	0.96	0.617	1.495
Q3	0.88	0.486	1.604	0.75	0.494	1.140
Q4	1.18	0.612	2.262	0.54	0.348	0.851
Supplemental vitamin C intake						
mg ^(2)^	1.00	0.989	1.005	1.00	0.992	1.002
Q1	Reference			Reference		
Q2	3.77	0.573	24.827	0.58	0.277	1.222
Q3	2.30	0.349	15.154	0.73	0.320	1.687
Q4	3.37	0.611	18.607	0.62	0.276	1.379
Dietary and supplemental vitamin C intake						
mg ^(2)^	1.00	0.991	1.005	1.00	0.991	1.001
Q1	Reference			Reference		
Q2	1.11	0.644	1.912	0.78	0.545	1.127
Q3	1.08	0.498	2.355	0.83	0.562	1.215
Q4	1.14	0.590	2.202	0.61	0.399	0.941

All analyses were conducted using sampling weights to reflect the complex sample design. Values are presented as Odds Ratios (ORs) and 95% Confidence Intervals (CIs). Multivariable logistic regression analysis was used to evaluate the risk of frailty. ^(1)^ Dietary vitamin C intake was adjusted for total energy intake using the residual method. ^(2)^ ORs are presented per 10-unit increase in the continuous variable.

**Table 4:** The risk of frailty by FV adequacy and vitamin C supplement use.

			Men			Women		
		% of Frail	OR	95% CI		OR	95% CI	
FV adequacy ^(1)^	FVA	3.02	Reference			Reference		
	FVINA	4.88	1.35	0.74	2.48	2.04	1.41	2.97
Vitamin C supplement use	Users	3.45	Reference			Reference		
	Non-users	4.54	1.57	0.87	2.85	1.11	0.80	1.54
FV adequacy and vitamin C supplement use ^(2)^	FVA non-supplement	3.2	Reference			Reference		
	FVA supplement	2.62	0.86	0.29	2.53	0.95	0.52	1.73
	FVINA supplement	3.97	0.79	0.35	1.80	1.88	1.10	3.21
	FVINA non-supplement	5.17	1.47	0.74	2.92	2.06	1.34	3.16

All analyses were conducted using sampling weights to reflect the complex sample design; data are presented in tables as weighted %. Values are presented as Odds Ratios (ORs) and 95% Confidence Intervals (CIs). Multivariable logistic regression analysis was used to evaluate the risk of frailty. Adjusted for age, single-person household status, household income, eating alone, education level, alcohol consumption, smoking status, diabetes, hypertension, and obesity, healthy eating index, energy intake and protein intake. FV, fruit and vegetable; FVA, fruit and vegetable adequacy; FVINA, fruit and vegetable inadequacy. ^(1)^ FV intake level was categorized as “FVINA (fruit and vegetable inadequacy)” if the total intake of fruits and vegetables was <500 g and “FVA (fruit and vegetable adequacy)” if it was ≥500 g. ^(2)^ “FVA non-supplement” refers to individuals who meet the ≥500 g/day fruit and vegetable intake but do not take supplements. “FVA supplement” refers to individuals who meet the ≥500 g/day fruit and vegetable intake and take supplements. “FVINA supplement” refers to individuals who consume < 500 g/day of fruits and vegetables and take supplements. “FVINA non-supplement” refers to individuals who consume < 500 g/day of fruits and vegetables and do not take supplements.

## Data Availability

This study analyzed data released from government agencies: https://knhanes.kdca.go.kr (accessed on 20 November 2025).

## Abbreviations

The following abbreviations are used in this manuscript:

FV	Fruit and vegetable
FVA	Fruit and vegetable adequate group
FVINA	Fruit and vegetable inadequate group
